# Geisinger MyCode^®^ detects BRCA2 mutation prior to abdominal panniculectomy allowing for DIEP flap breast reconstruction

**DOI:** 10.1080/23320885.2019.1684824

**Published:** 2019-11-06

**Authors:** Christian A. Kauffman

**Affiliations:** Department of Plastic Surgery, Geisinger Medical Center, Danville, PA, USA

**Keywords:** Breast reconstruction, mastectomy, panniculectomy, abdominoplasty, deep inferior epigastric perforator flap

## Abstract

This case report presents a 38-year-old female who was scheduled for abdominal panniculectomy and tested positive for BRCA2 mutation through Geisinger MyCode^®^. She canceled her panniculectomy and elected to proceed with bilateral mastectomies, utilizing her abdominal tissue for deep inferior epigastric perforator flap reconstruction.

## Introduction

Geisinger MyCode^®^ is a system-wide biorepository of blood, serum and DNA samples which allows for genomic analysis [1]. Geisinger Health System is in central and northeastern Pennsylvania and New Jersey, with a unique patient population due to its relative stability and multi-generational families [1]. The MyCode^®^ project began in 2007 with a goal of performing genetics research on a stable patient population because the genomics data could be combined with the electronic health record. The research was not focused on one set of genetic mutations or diagnoses, but it was being used with the intention of detecting more genetic mutations for future patients. Patient participation is entirely voluntary and has continued to grow with over 180,000 patients enrolled as of January 2018. As more data were collected, it became apparent that patients could benefit from knowing some of the results from the genetic testing, even though this was not the primary purpose of the project. In 2013, a change was made to the protocol which allowed for patients to be contacted if the results were medically actionable [1]. These types of results would be placed in the medical record, and the physicians would be contacted. Examples of these results include hereditary breast and ovarian cancer, familial hypercholesterolemia, Lynch syndrome, cardiomyopathy, arrhythmia, arrhythmogenic right ventricular cardiomyopathy, Marfan syndrome, heritable thoracic aortic disease, hereditary pheochromocytomas and paragangliomas, multiple endocrine neoplasia type 1 and 2, PTEN hamartoma tumor syndrome, tuberous sclerosis, Li-Fraumeni syndrome, Familial adenomatous polyposis, Von Hippel-Lindau, malignant hyperthermia, Fabry disease, vascular Ehlers-Danlos, and hereditary hemochromatosis. As of January 1, 2018, 544 patient-participants have received results there were medically actionable. Two hundred and three patients (37%) tested positive for BRCA 1 (68) or BRCA 2 (135) mutation. The BRCA1 mutation increases the chance of developing breast cancer to 55–65% and BRCA2 mutation increases the chance of developing breast cancer to 45%. For patients with medically actionable results, they are notified of the positive result and then referred to the appropriate specialists to discuss treatment options.

For patients who are found to have a BRCA1 or BRCA2 mutation but do not have breast cancer, they have the option of bilateral prophylactic mastectomies or close surveillance. Bilateral prophylactic mastectomies provide the most protection from developing breast cancer with an 87% risk reduction [2]. The rates of breast reconstruction after prophylactic mastectomy in BRCA1 and BRCA2 mutation carriers were reported in 2013, and approximately 72% of patients in the United States had breast reconstruction following prophylactic mastectomy, whether unilateral with a diagnosis of breast cancer or bilateral with no diagnosis of breast cancer [2]. Younger women are more likely to undergo breast reconstruction with options that include implant-based reconstruction or autologous reconstruction [2]. The abdomen is the most common location to obtain tissue for autologous reconstruction, utilized as either a free transverse abdominis myocutaneous (TRAM) or deep inferior epigastric perforator (DIEP) flap. Prior abdominoplasty or abdominal surgery that destroyed the flap perforators has been considered a contraindication to TRAM and DIEP flap reconstruction [3,4], There are some reports of successful free TRAM flap and DIEP flap breast reconstruction after abdominoplasty, although it is still not generally recommended [4–6].

This case report presents a 38-year-old female who had surgery scheduled for a lower abdominal panniculectomy, but while awaiting surgery, was found to have a BRCA2 mutation. Her decision and subsequent treatment is described.

## Material and methods

Chart review was performed of a 38-year-old female who was initially evaluated for symptomatic abdominal panniculectomy. She had a history of laparoscopic sleeve gastrectomy 12 months prior which resulted in a 120-pound weight loss. She was scheduled to have a lower abdominal panniculectomy, and soon after scheduling she entered the Geisinger MyCode^®^ program. Despite having no family history of breast cancer, she tested positive for BRCA2 mutation four weeks prior to surgery. Discussion with the patient included risk of breast cancer, reconstruction options, and that the lower abdominal panniculectomy would limit the use of her abdominal tissue for an autologous reconstruction. The surgery was cancelled and after additional consultations with a genetic counselor and a breast surgeon, she decided to proceed with bilateral skin sparing mastectomies and immediate-delayed autologous reconstruction by initially placing tissue expanders. Her post-operative course was uncomplicated, and her tissue expanders were expanded to 450 cc’s. Five months after her mastectomies, she had bilateral breast reconstruction with DIEP flaps.

## Results

She had a successful bilateral breast reconstruction with DIEP flaps after discovery of BRCA2 mutation. Ten months post-operatively, she remains very happy with her decision to enter the MyCode^®^ program, have bilateral mastectomies and reconstruction with bilateral DIEP flaps.

## Discussion

This case illustrates another significant benefit that genomic analysis can provide, even for those patients without a family history of breast cancer. Genetic testing is typically used in patients who have a strong family history of breast cancer; however, this patient would not have had genetic testing if the classic indications were used. By participating in this initiative, she was warned about her increased risk of breast cancer prior to developing breast cancer, and she was able to have all her breast reconstructive options available by cancelling her lower abdominal panniculectomy. The MyCode^®^ results allowed her to make decisions about her healthcare proactively instead of reactively. As genetic research continues, and more diseases and syndromes are identified, the potential benefits for our patients will increase exponentially. To date, hundreds of patients have been granted the power to make more informed medical decisions based on their genetic results. The MyCode^®^ project will continue to expand its value as more participants enroll over time, allowing for greater population analysis which should result in detection of additional diseases. As more patients are notified of their actionable results, this truly defines preventative medicine as it allows patients to intervene in their healthcare prior to development of disease.

This case also highlights an important issue regarding informed consent for patients undergoing panniculectomy or abdominoplasty. Discussing the potential loss of abdominal tissue for future reconstruction should be part of any thorough informed consent. Unfortunately, 87% of surgeons did not routinely consent patients regarding the loss of TRAM flap as a reconstructive option after abdominoplasty [7]. Additionally, 83 preprinted abdominoplasty consent forms were obtained from hospitals worldwide, and none of the forms discussed that abdominoplasty would limit potential reconstructive options [8]. These authors advocate for routinely informing female patients who are going to have an abdominoplasty about the potential limitations for future breast reconstruction [7,8]. Even though we know that this should be part of the informed consent, we must all be more vigilant in discussing this downside of panniculectomy and abdominoplasty with our patients. Despite reports of successful DIEP or TRAM flap reconstruction after abdominoplasty, most patients who have an abdominoplasty or panniculectomy would be limited in their reconstructive options. Due to the frequency that breast cancer is diagnosed, it is imperative that we inform our patients regarding how their decisions can impact future options regarding breast reconstruction after abdominoplasty or panniculectomy.

This case report highlights the potential benefits for the individual patient who chooses to enroll in the MyCode^®^ project. It is important to note that the MyCode^®^ project is not meant to replace genetic testing for those individuals at increased risk for breast cancer based on family history. While the overall goal of the project is as a precision medicine health initiative for DNA analysis of enrolled individuals for the benefits of future patients, there has been an initial side benefit for patients who have actionable results detected. The data will continue to be more useful as the number of participants increases and continued research discovers new diseases or syndromes identifiable through genomics. Our awareness of how genomics can impact our patients will allow us to better counsel them regarding their treatment options, ultimately leading to better overall care.
